# Genetic intraspecific diversity of *Meloidogyne javanica* parasitizing vegetables in southern Iran

**DOI:** 10.21307/jofnem-2020-035

**Published:** 2020-04-24

**Authors:** Reza Ghaderi, Ali Asghar Dehghan, Abbas Mokaram Hesar, Akbar Karegar

**Affiliations:** 1 Department of Plant Protection, School of Agriculture, Shiraz University, 71441-65186, Shiraz, Iran

**Keywords:** Diversity, ISSR, RAPD, root-knot nematodes, species-specific primer

## Abstract

In order to investigate different species of root-knot nematodes associated with vegetable production in southern regions of Iran, 37 populations of the most predominant species, *Meloidogyne javanica*, were recovered. Morphological and morphometric studies were carried on the characters of females, males, J2s and perineal patterns. Species-specific Sequence Characterized Amplified Region (SCAR) primers confirmed morphological studies, and all these populations produced specific band in 670 bp using Fjav and Rjav primers. Genetic diversity of different populations was studied by Inter Simple Sequence Repeats (ISSR) and Random Amplified Polymorphic DNA (RAPD) markers implementing 10 primers for each approach. Results revealed a relatively low genetic diversity (the percentage of polymorphic bands were 19.08 and 24.60 for ISSR and RAPD, respectively). The analyses of molecular variance indicated that the variation resulted from genotypic variations within region and variances among regions are 81% and 19% for RAPD, and 86% and 14% for ISSR, respectively. On the other hand, *F*_*ST*_ and Nm values are 0.140 and 1.535 for ISSR while these values are 0.188 and 1.079 for RAPD. So it can be concluded that there is a great deal of gene flow between populations due to the movement of plant material contaminated with nematodes, which results in high mixing between populations. ISSR and RAPD datasets failed to group populations according to their geographic region. There were no pathotypes or other intraspecific biological entities observed in the species.

Plant-parasitic nematodes are an important limiting factor in vegetable production, and in many areas a major factor requiring extensive use of pesticides. Root-knot nematodes (RKNs) of the genus *Meloidogyne* (Göldi, 1887), which their population increase to damaging levels within a few seasons under susceptible crops, are so common in subtropical and tropical vegetable production that frequently they are taken to represent “nematodes” in general (Sikora and Fernandez, 2005). The populations of *M. incognita* (Kofoid and White, 1919) Chitwood, 1949 and *M. javanica* (Treub, 1885) Chitwood, 1949 were the most recovered populations (more than 80%) from vegetable fields worldwide (Taylor and Sasser, 1978). Five species including *M. arenaria* (Neal, 1889) Chitwood, 1949, *M. cruciani* Garcia-Martinez, 1982, *M. hapla* Chitwood, 1949, *M. incognita* and *M. javanica* are the recovered RKN in vegetable production in Iran (Mahdikhani Moghadam, 2018).

Considering that morphological and morphometrical methods for the identification species of RKNs are considerably time consuming and there is a lot of ambiguity, using species-specific primers could be a rapid method for detection (these primers listed in Cunha et al., 2018).

The genetic diversity among *M. javanica* populations has been inferred by different markers (reviewed in Blok and Powers, 2009; Mokaram Hesar et al., 2011). Statistical analysis of the morphological characters and RAPD markers demonstrated 86 and 93% similarity among 21 populations of *M. javanica* recovered from north east of Iran. Intraspecific variation was less influenced by geographical origin of nematodes and/or their host plants (Mokaram Hesar et al., 2011). RAPD-PCR technique was utilized successfully to differentiate among three important RKN species, *M. arenaria*, *M. incognita*, and *M.  avanica*, and also, to evaluate genetic variations among different populations of *M. javanica* (Naz et al., 2013). RAPD markers revealed a low intraspecific genetic diversity (14%) among 17 isolates of this species parasitizing potato in Brazil (Medina et al., 2017). Principal component and phylogenetic analyses revealed distant relationship between *M. javanica* and *M. enterolobii* Yang and Eisenback, 1983 populations from South Africa (Rashidifard et al., 2018).

Molecular markers that have proved to be useful in these studies are mitochondrial gene cytochrome C oxidase subunit I (COI) (Ye et al., 2015; Powers et al., 2018), the small subunit 18 S rRNA gene (Tigano et al., 2005; Ye et al., 2015), D2-D3 expansion segment of the large subunit 28 S rRNA (Palomares-Rius et al., 2007; Ye et al., 2015) and ribosomal internal transcribed spacer (ITS1 and ITS2) (Powers et al., 1997; Ye et al., 2015). These markers were useful for separating species of RKNs; however, they could not separate main species including *M. javanica*, *M. incognita*, *M. hapla*, and *M. arenaria*, and there is overlapping between these species (Kiewnick et al., 2014).

Although ISSR technique has been used to resolve genetic diversity in certain species of RKNs such as *M. enterolobii* (Tigano et al., 2010) and *M. arenaria* (Carneiro et al., 2008), it has not been implemented for *M. javanica* so far.

However, ISSR and RAPD markers are similar in some aspects, but there are a number of differences between them that make the two markers act as complementing of each other. RAPD primers are short (10 nucleotides) with a random sequence and markers are considered to be uniformly distributed along the genome, whereas ISSR primers is based on the amplification of regions flanked by repeating sequences which found only between microsatellite loci. In the other hands, annealing temperature in RAPD is lower than ISSR (Lax et al., 2007).

The aim of the present study is to infer the genetic intraspecific diversity among populations of *M. javanica* collected from major vegetable growing areas in southern regions of Iran, using RAPD and ISSR markers.

## Material and methods

### Nematode populations and light microscopy

In total, 37 populations of *M. javanica* were collected from vegetables (tomato, cucumber, okra, and eggplant) and weeds of vegetable fields in several regions of southern Iran, during 2018 and 2019 (Table 1). Nearly 700 fields and greenhouses were sampled, randomly or sometimes from infested patches of affected plants showing symptoms, in several times during 2017 and 2018 years. In total, 57 populations were recovered on roots of crops such as tomato, cucumber, eggplant, and okra in different regions of Fars, Boushehr, Kerman, and Kohgiloyeh and Boyer-Ahmad provinces. Pure cultures of all populations were produced by rearing a single egg mass on tomato (cv. Early Urbana) grown in sterilized river sand in clay pot, at a temperature ranging 20 to 30°C. After 70 days, mature females were extracted from stained roots for further morphometric analysis of the females and preparing perineal patterns. Second-stage juveniles (J2s) and males, if present, were killed and fixed by hot FPG (4:1:1, formaldehyde: propionic acid: glycerin), and processed to anhydrous glycerin (de Grisse, 1969). The J2s, males, and perineal patterns were then mounted on permanent slides and examined for morphological and morphometric purposes using an Olympus BX41 light microscope, equipped with Dino-eye microscope eye-piece camera and its Dino Capture version 2.0 software.

**Table 1.** Populations of *Meloidogyne* species collected in this study.

Number	Sampling area	N	E	Sample	Host plant	Molecular code
1	Boshehr province-Abdan	28°11´00.38˝	51°74´07.94˝	Soil and root	Tomato	MJ1
2	Boshehr province-Abdan	28°10´99.86˝	51°74´08.46˝	Soil and root	Tomato	MJ2
3	Boshehr province-Abdan	28°11´00.58˝	51°74´07.97˝	Soil and root	Tomato	MJ3
4	Boshehr province-Abdan	28°11´00.29˝	51°74´08.27˝	Soil and root	Tomato	MJ4
5	Boshehr province-Abdan	28°11´00.31˝	51°74´08.28˝	Soil and root	Tomato	MJ5
6	Boshehr province-Abdan	28°10´95.09˝	51°74´04.66˝	Soil and root	Tomato	MJ6
7	Boshehr province-Abdan	28°10´94.93˝	51°74´04.44˝	Soil and root	Tomato	MJ7
8	Boshehr province-Abdan	28°10´94.82˝	51°74´04.43˝	Soil and root	Tomato	MJ8
9	Boshehr province-Abdan	28°10´94.56˝	51°74´03.85˝	Soil and root	Tomato	MJ9
10	Boshehr province-Abdan	28°10´94.47˝	51°74´03.81˝	Soil and root	Tomato	MJ10
11	Boshehr province-Deir Port	27°91´15.43˝	51°98´02.27˝	Soil and root	Tomato	MJ11
12	Boshehr province-Deir Port	27°91´15.16˝	51°98´02.71˝	Soil and root	Tomato	MJ12
13	Boshehr province-Deir Port	27°91´17.39˝	51°98´03.58˝	Soil and root	Tomato	MJ13
14	Boshehr province-Deir Port	27°91´17.33˝	51°98´03.47˝	Soil and root	Tomato	MJ14
15	Boshehr province-Deir Port	27°91´44.84˝	51°98´43.36˝	Soil and root	Tomato	MJ15
16	Boshehr province-Bardkhon	28°07´54.24˝	51°48´89.26˝	Soil and root	Tomato	MJ16
17	Boshehr province-Bardkhon	28°07´51.78˝	51°48´90.21˝	Soil and root	Tomato	MJ17
18	Boshehr province-Bardkhon	28°36´13.93˝	51°48´90.22˝	Soil and root	Tomato	MJ18
19	Boshehr province-Bardkhon	28°07´51.25˝	51°48´84.44˝	Soil and root	Tomato	MJ19
20	Boshehr province-Bardkhon	28°07´51.92˝	51°48´82.48˝	Soil and root	Tomato	MJ20
21	Boshehr province-Bardkhon	28°07´52.13˝	51°48´82.42˝	Soil and root	Tomato	MJ21
22	Boshehr province-Bardkhon	28°07´52.15˝	51°48´82.19˝	Soil and root	Tomato	MJ22
23	Kerman province-Jiroft	28°41´09.00˝	57°40´13.81˝	Root	Mallow	MJ23
24	Kerman province-Jiroft	28°41´35.08˝	57°39´25.18˝	Root	Goosefoot	MJ24
25	Kerman province-Jiroft	28°42´22.57˝	57°43´05.86˝	Root	Eggplant	MJ25
26	Fars province-Beyza	29°55´30.53˝	52°26´28.41˝	Soil and root	Cucumber	MJ26
27	Fars province-Beyza	29°55´51.85˝	52°26´44.88˝	Soil and root	Cucumber	MJ27
28	Fars province-Beyza	29°55´59.49˝	52°26´51.00˝	Soil and root	Cucumber	MJ28
29	Fars province-Beyza	29°55´16.00˝	52°27´20.15˝	Soil and root	Cucumber	MJ29
30	Fars province-Beyza	29°55´11.72˝	52°27´27.93˝	Soil and root	Cucumber	MJ30
31	Fars province-khafr	28°57´44.57˝	53°11´58.46˝	Soil and root	Okra	MJ31
32	Fars province-khafr	28°57´35.80˝	53°12´32.31˝	Soil and root	Okra	MJ32
33	Fars province-khafr	28°57´34.97˝	53°12´28.38˝	Soil and root	Okra	MJ33
34	Fars province-khafr	28°58´56.08˝	53°11´47.87˝	Soil and root	Okra	MJ34
35	Fars province-Kazeron	29°33´23.23˝	51°45´56.27˝	Soil and root	Tomato	MJ35
36	Fars province-Kazeron	29°33´25.22˝	51°45´42.76˝	Soil and root	Tomato	MJ36
37	Fars province-Kazeron	29°33´42.68˝	51°45´08.64˝	Soil and root	Tomato	MJ37
38	Fars province-Fasa	29°00´46.56˝	53°37´42.27˝	Soil and root	Cucumber	MI1
39	Fars province-Fasa	29°00´46.56˝	53°37´42.27˝	Soil and root	Cucumber	MI2
40	Fars province-Fasa	29°00´46.56˝	53°37´42.27˝	Soil and root	Cucumber	MI3
41	Fars province-Fasa	29°00´46.56˝	53°37´42.27˝	Soil and root	Cucumber	MI4

**Notes:** MJ: *M. javanica*; MI: *M. incognita.*

### DNA extraction

One full egg mass was put in 16 μl ddH_2_O in a 0.2 ml tube. DNA was extracted according to Tanha Maafi et al. (2003) with some modifications: tubes were frozen at –80°C for at least 15 min and crushed by vortexing, and 20 μl worm lysis buffer (500 mM KCl, 100 mM Tris-Cl pH 8, 15 mM MgCl_2_, 0.05% Mercaptoethanol, and 4.5% Tween 20) and 4 μl proteinase K (600 μg/ml) were added, respectively. The samples were incubated at 65°C for 1 hr and at 95°C for 10 min. After incubation, the tubes were centrifuged for 2 min at 38,000 g and kept at –20°C for next uses.

### PCR using species-specific primers

PCR was performed containing 1 μl of DNA template, 8 μl mastermix (Amplicon Red), 1 μl of each primer (10 pmol) and 4 μl ddH2O to a final volume of 15 μl. Four set species-specific primers (Table 2) were used for PCR reactions (Zijlstra, 2000; Zijlstra et al., 2000). A negative control containing the PCR mixture without DNA template was also included.

**Table 2.** All primers used for ISSR, RAPD and species-specific identification of *Meloidogyne* species during the present study.

ISSR		RAPD	
(CCA)_5_	5´-CCACCACCACCACCA-3'	OPA8	5’-GTGACGTAGG-3'
(GTG)_6_	5'-GTGGTGGTGGTGGTGGTG-3'	OPA10	5'-GTGATCGCAG-3'
(ATG)_6_	5'-ATGATGATGATGATGATG-3'	OPA13	5'-CAGCACCCAC-3'
(GAG)_4_GC	5'-GAGGAGGAGGAGGC-3'	OPAD10	5'-AAGAGGCCAG-3'
(GACA)_4_	5'-GACAGACAGACAGACA-3'	OPE18	5'-GGACTGCAGA-3'
(GA)_8_C	5'-GAGAGAGAGAGAGAGAC-3'	OPP17	5'-TGACCCGCCT-3'
(AC)_8_T	5'-ACACACACACACACACT-3'	OPB11	5'-GTAGACCCGT-3'
(CA)_8_G	5'-CACACACACACACACAG-3'	OPB17	5'-AGGGAACGAG-3'
(CTC)_4_GC	5'-CTCCTCCTCCTCGC-3'	OPD8	5'-GTGTGCCCCA-3'
(GAGA)_4_GG	5'-GAGAGAGAGAGAGAGAGG-3'	OPC8	5'-TGGACCGGTG-3'
*Species-specific primers*			
*M. javanica*	Fjav (5'-GGTGCGCGATTGAACTGAGC-3')	*M. incognita*	Finc (5'-CTCTGCCCAATGAGCTGTCC-3')
	Rjav (5'-CAGGCCCTTCAGTGGAACTATAC-3')		Rinc (5'-CTCTGCCCTCACATTAAG-3')
*M. arenaria*	Far (5'-TCGGCGATAGAGGTAAATGAC-3')	*M. hapla*	F (5'- TGACGGCGGTGAGTGCGA-3')
	Rar (5'-TCGGCGATAGACACTACAAACT-3')		R (5'-TGACGGCGGTACCTCATAG-3')

The PCR amplification profile consisted of 5 min at 94°C, 35 cycles of 30 sec at 94°C, 30 sec at annealing temperature (64°C for *M. javanica*, 54°C for *M. incognita*, 61°C for *M. arenaria*, and 58°C for *M. hapla*) and 45 sec at 72°C, followed by a final step of 5 min at 72°C. All steps were repeated twice to insure the results. PCR success was visualized loading 4 μl of the PCR product into a 1.7% TBE buffered agarose gel (75 V, 60 min) and visualized with ethidium bromide.

### ISSR-PCR

The ISSR-PCR reactions were performed in a 20 μl volume containing 2 μl of genomic DNA, 2 μl of primer, 8 μl mastermix (Amplicon Red), and 8 μl ddH_2_O. Ten primers including (CCA)_5_, (GTG)_6_, (ATG)_6_, (GAG)_4_GC, (GACA)_4_, (GA)_8_C, (AC)_8_T, (CA)_8_G, (CTC)_4_GC, and (GAGA)_4_GG (Table 2) were implemented (Lax et al., 2007).

PCR ampliﬁcation reactions were programmed for an initial denaturation at 94°C for 3 min, followed by 35 cycles of 30 sec at 93°C, 90 sec at 48°C, 1 min at 72°C, and a ﬁnal extension of 10 min at 72°C. PCR success was visualized by loading 7 μl of the PCR product into a 1.7% TBE buffered agarose gel (75 V, 120 min).

### RAPD-PCR

Amplification reactions were carried out in reactions of 20 μl total volume containing 2 μl of genomic DNA, 2 μl of primer, 8 μl mastermix (Amplicon Red), and 8 μl ddH_2_O. Ten primers (Table 2) were used in RAPD-PCR (Mokaram Hesar et al., 2011).

For RAPD amplifications, the thermos-cycle profile was 5 min at 94°C; 35 cycles of 1 min at 94°C, 1 min at 35°C and 2 min at 72°C, followed by a final step of 5 min at 72°C. PCR success was visualized loading 7 μl of the PCR product into a 1.7% TBE buffered agarose gel (75 V, 120 min).

### ISSR and RAPD data analysis

For the ISSR and RAPD fragment analysis, data were scored for all primers: “1” for the presence of band and “0” for the absence of band. To be sure, all steps were repeated twice and smeared or weak bands were not counted. Distance matrixes were calculated using the Dice and Jaccard coefficients. Cluster analysis was performed with the Numerical Taxonomy Multivariate Analysis System (NTSYSPC V-2.02) software package (Rohlf, 2000). The genetic dissimilarity matrix and ultrametric distance matrix produced from UPGMA-based dendrogram with COPH module nested in the same software was compared using Mantel’s matrix correspondence test (Mantel, 1967). Also, we try to use Mantel test in linear shape for searching correlation between different matrix produced by RAPD and ISSR. Bootstrapping was done using the Winboot with 1000 replicates (Yap and Nelson, 1996) and measurements under 60 were excluded. Frequency analyses were performed to estimate the variance components and their significance levels of genetic variation within and among populations using GenALEx version 6.5 (Peakall and Smouse, 2006). We used P (percentage of polymorphic loci), H (the expected heterozygosity) and I (Shannon’s information index) to calculate genetic diversity. Also, *F*_*ST*_ (Fixation index) and Nm (Number of migrants) used to calculate the differences of populations and amount of gene flow between populations. These two indices are inversely correlated, and by increasing fixation index, Nm is decreased (Milgroom, 2015).

## Results

### Characterization of the recovered populations

**Figure 1:**
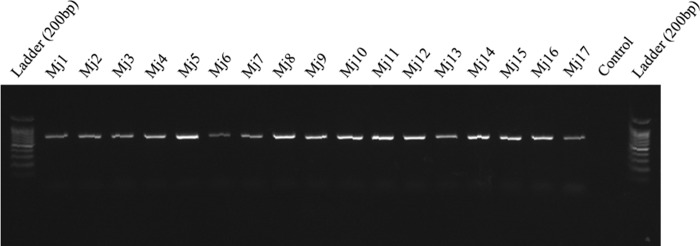
Species-specific 670 bp band in the populations of *Meloidogyne javanica* recovered from southern Iran. (For population codes, see Table 1).

**Figure 2:**
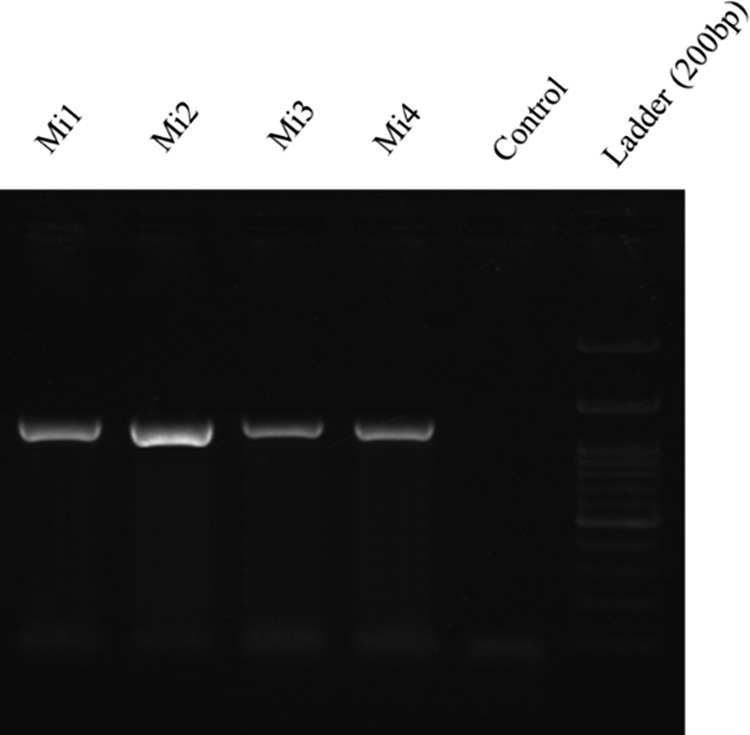
Species-specific 1200 bp band in the populations of *Meloidogyne incognita* recovered from southern Iran. (For population codes, see Table 1).

In total, 41 populations of RKNs were recovered including 34 populations from vegetable fields and nine populations from greenhouses in southern Iran. Two nematodes populations were obtained from weeds growing in vegetable fields. All the recovered populations in this study were tested with the species-speciﬁc molecular marker type Sequence Characterized Amplified Region (SCAR) for the four major species (Zijlstra, 2000; Zijlstra et al., 2000). Expected band for *M. javanica*, *M. incognita*, *M. arenaria*, and *M. hapla* are 670 bp, 1200 bp, 420 bp, and 960 bp, respectively. The species-specific primers of *M. javanica* (Fjav and Rjav) yielded one unique fragment of approximately 670 bp in 37 populations, and no PCR product was obtained in the control template (due to the limitation of the number of wells in electrophoresis, the PCR products were run separately, and the results of 25 populations were presented in Fig. 1). Also, no bands were detected in the gels of the 37 isolates of other three species (data not presented). The primers of *M. incognita* yielded a 1,200 bp band for four isolates of greenhouse cucumber in Fasa region (Fig. 2). Morphometric characters for the females, J2s, and males of some populations of *M. javanica* are given in Table 3.

**Table 3.** Average of the morphometrics of females, J2s and males of five populations of *Meloidogyne javanica* parasitizing vegetables in southern Iran.

	Females		Males		Second-stage juveniles (J2s)^†^						
Diagnostic characters	15	CV	10	CV	MJ1	MJ4	MJ8	MJ14	MJ15	50	CV
L (including neck)	686±127 (506-1,010)	18	1482±155 (1189-1,694)	10	463	457	449	482	469	464±19 (429-497)	4
Body width (W)	420±99 (274-568)	23	33±2.8 (29-38)	9	14	14	15	15	16	15±1.5 (12-18)	9
Neck length	259±87 (153-540)	33	−	−	−	−	−	−	−	−	−
Stylet	15±2 (13-18)	14	18±1.2 (16-19)	6	9.7	10.4	10.5	10.1	10.2	10±0.5 (9-12)	5
DGO	3.1±0.6 (2.0-3.9)	20	3.3±0.2 (3.1-3.5)	5	2.7	2.1	2.2	1.9	2.0	2.2±0.5 (1.5-3.7)	22
Excretory pore	190±30 (145-270)	16	−	−	−	−	−	−	−	−	−
Vulval slit length	21±3 (15-27)	15	−	−	−	−	−	−	−	−	−
Vulva-anus distance	16±4 (12-28)	25	−	−	−	−	−	−	−	−	−
Inter-phasmidial distance	78 ± 16 (58-108)	20	−	−	−	−	−	−	−	−	−
Tail length	−	−	14±2.1 (10-17)	15	51	55	55	58	57	55±5 (40-66)	9
Hyaline length	−	−	−	−	11	12	11	16	16	13±4 (7-20)	27
Pharynx (glands end)	−	−	473±50 (390-552)	11	164	202	197	201	201	193±23 (90-216)	12
Body length to width (a)	1.7±0.4 (1.2-2.6)	22	45±4.5 (37-51)	10	38	32	29	33	30	31±3 (25-39)	10
b	−	−	6.6±1.3 (4.8-8.1)	20	6.4	4.9	4.7	5.0	5.2	5.2±1.4 (4.3-12.4)	27
b'	−	−	3.1±0.6 (2.4-3.9)	18	3.0	2.3	2.3	2.4	2.3	2.5±0.5 (2.0-5.2)	22
c	−	−	113±26 (87-169)	23	9.4	8.4	8.2	8.3	8.2	8.5±0.8 (7.2-11.3)	10
c'	−	−	0.5±0.1 (0.3-1.0)	19	4.6	5.3	5.5	5.9	5.2	5.7±0.5 (4.7-7.2)	10
Spicules	−	−	23±5.8 (15-29)	24	−	−	−	−	−	−	−
Gubernaculum	−	−	13±2.8 (8-17)	20	−	−	−	−	−	−	−

**Notes:** Measurements are in μm. Data are the average of the measurements. ^†^10 specimens were measured from each population.

### Inferring genetic diversity by RAPD and ISSR markers

**Figure 3:**
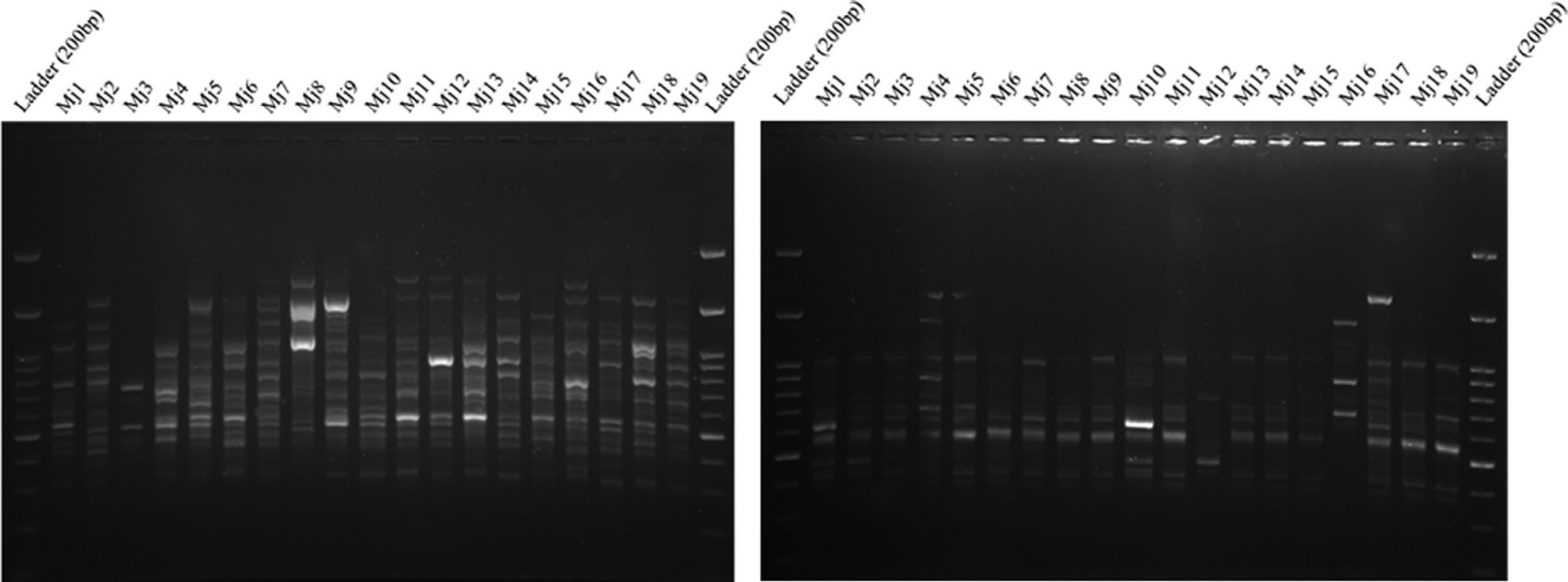
RAPD (left) and ISSR (right) patterns for 19 populations of *Meloidogyne javanica* generated with the primer (CCA)_5_ and OPAD10. DNA Ladder = 200 bp. (For population codes, see Table 1).

With the 10 random primers used in the RAPD analysis, a total of 180 bands were produced. All primers resulted in ampliﬁcation and different patterns were tested for population differences. The resulting gel electrophoresis for 19 populations for (CCA)_5_ primer of ISSR and OPAD10 primer of RAPD is presented in Figure 3. The number of reproducible ampliﬁed fragments varied from 14 to 23 per primer (average 18), their size ranging from 200 to 2500 bp. The percentage of polymorphic bands (P%) within each population ranged from 16.1 to 36.1%, (in populations of Khafr and Abdan, respectively) and I index ranged from 0.093 to 0.218 (same as P%). The highest value of H index was in populations of Abdan (0.150). The mean gene variations for the 27 populations were 0.103 and 0.148 by H and I, respectively (Table 4).

**Table 4.** Genetic diversity estimates of *Meloidogyne javanica* populations recovered from southern Iran by RAPD and ISSR analyses.

	ISSR			RAPD		
Population	%P^†^	H	I	%P	H	I
Abdan (10)^††^	25.61	0.110	0.158	36.11	0.150	0.218
Deir (5)	20.73	0.092	0.130	30.00	0.132	0.189
Bardkhon (7)	24.39	0.097	0.141	30.56	0.118	0.173
Jiroft (3)	10.37	0.039	0.058	17.78	0.079	0.113
Beiza (5)	21.34	0.088	0.128	22.78	0.095	0.137
Khafr (4)	18.29	0.076	0.110	16.11	0.064	0.093
Kazeron (3)	12.80	0.057	0.081	18.89	0.079	0.114
Mean	19.08	0.080	0.115	24.60	0.103	0.148

**Notes:** ^†^P(%): percentage of polymorphic loci; H: the average expected heterozygosity; I: Shannon’s information index; ^††^the data between parentheses are the number of populations collected from the given region.

In ISSR, a total of 164 loci, with average of 16.4 loci per primer, were scored. P, H, and I were used to estimate genetic diversity within the *M. javanica* populations. The populations of Abdan and Bardkhon exhibited the highest level of genetic diversity (P% = 25.6, H = 0.110, I = 0.158; P% = 24.39, H = 0.097, I = 0.141, respectively), whereas the lowest value was shown in the populations of Jiroft (P% = 10.37, H = 0.039, I = 0.058). In 164 loci, 19.08% were polymorphic in all individuals of studied populations. The mean genetic variations of total indicated by H and I, were 0.080 and 0.115, respectively.

The Nei’s (1978) genetic distances (D) between pairs of *M. javanica* populations with RAPD analysis were 0.043 to 0.162 (Table 5). The lowest value was between the populations of Abdan and Beiza, and the highest between the populations of Kazeron and Khafr. While for ISSR analysis, pairwise D values were ranged from 0.032 (populations of Abdan and Deir) to 0.091 (populations of Kazeron and Deir, also Abdan and Beiza). Mantel test showed a positive correlation between RAPD and ISSR (Fig. 6) and an increasing on each marker values was observed with an increasing on another marker values.

**Table 5.** **N**ei’s (1978) unbiased genetic distance between pairs of *Meloidogyne javanica* populations detected by RAPD and ISSR analyses.

Population^†^	Abdan	Deir	Bardkhon	Jiroft	Beiza	Khafr	Kazeron
Abdan	–	0.032	0.070	0.074	0.091	0.084	0.088
Deir	0.082	–	0.054	0.076	0.068	0.088	0.091
Bardkhon	0.090	0.076	–	0.046	0.045	0.039	0.059
Jiroft	0.069	0.127	0.119	–	0.071	0.040	0.025
Beiza	0.043	0.080	0.119	0.108	–	0.073	0.071
Khafr	0.080	0.055	0.143	0.134	0.075	–	0.051
Kazeron	0.120	0.071	0.050	0.156	0.132	0.162	–

**Note:** ^†^Nei’s genetic distance matrices detected by RAPD (above diagonal) and ISSR (below diagonal).

**Figure 4:**
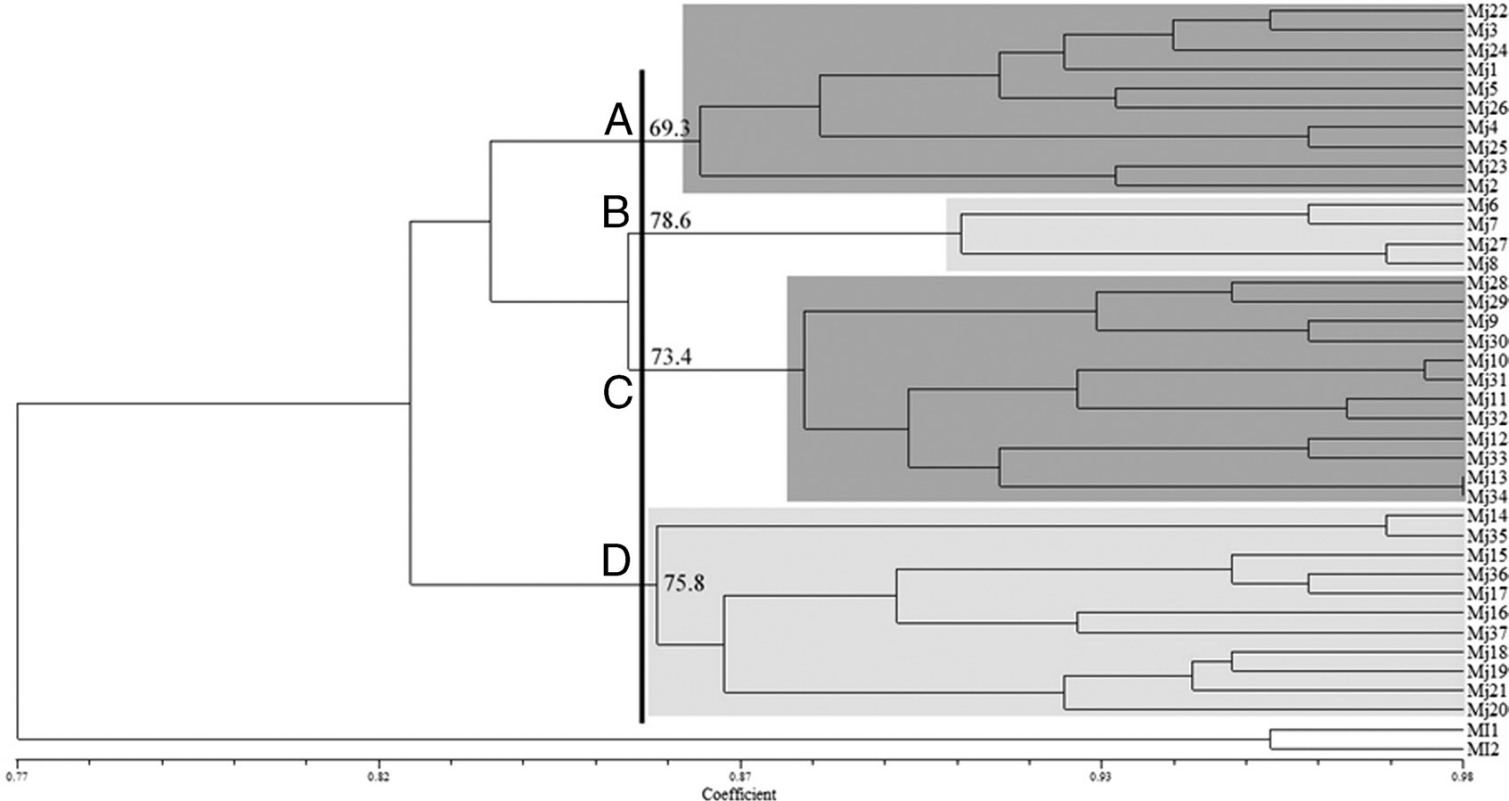
UPGMA cluster diagram based on Dice coefficient estimated from 180 RAPD markers for 37 populations of *Meloidogyne javanica* (For population codes, see Table 1).

**Figure 5:**
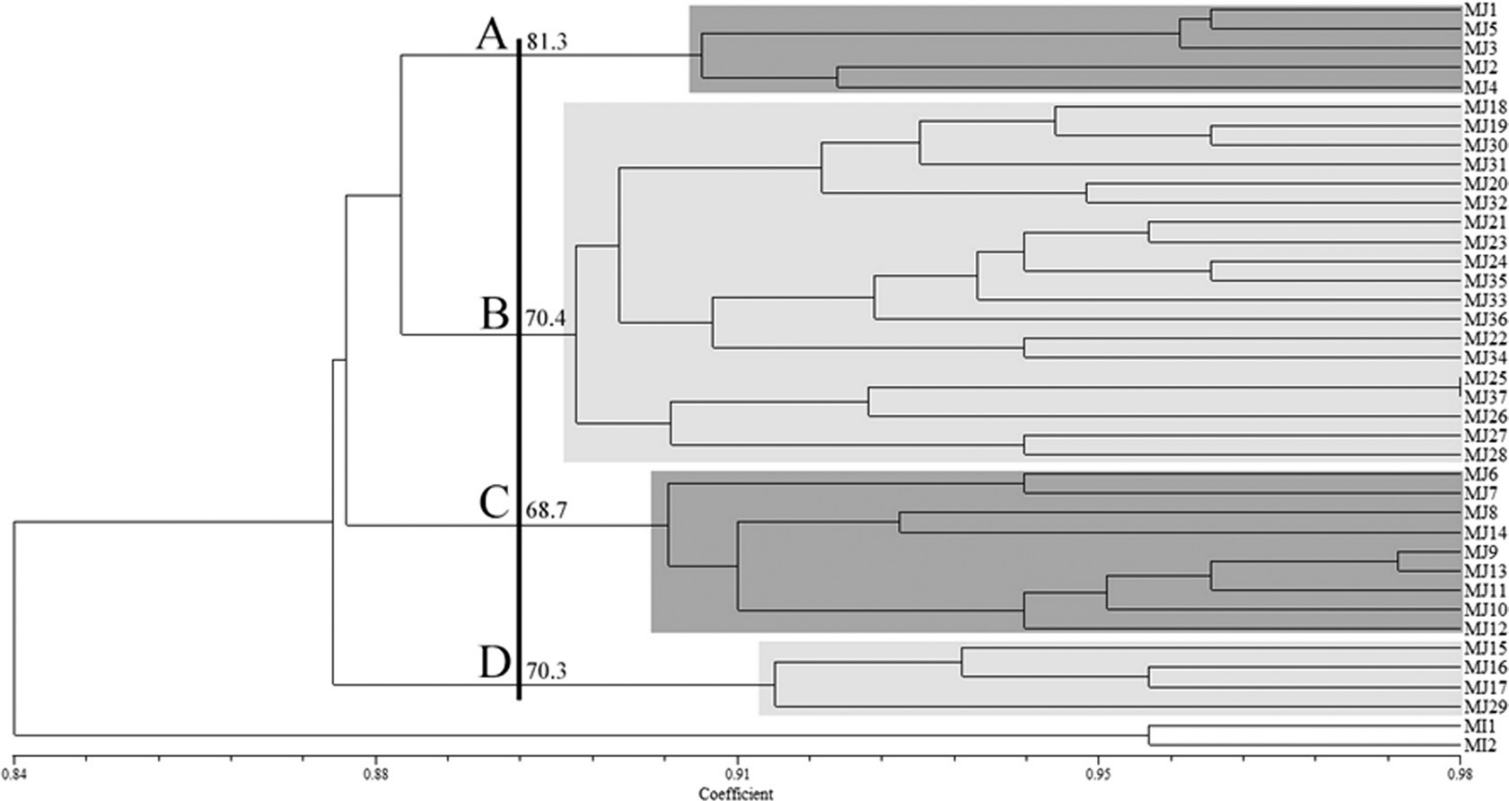
UPGMA cluster diagram based on Dice coefficient estimated from 164 ISSR markers for 37 populations of the *Meloidogyne javanica* (For population codes, see Table 1).

The UPGMA trees obtained with RAPD and ISSR data showed some differences in tree topologies between calculations based on Dice and Jaccard. The positions of some populations are different by ISSR and RAPD, although the number of clades was consistent as both methods revealed four clades. None of these clades grouped *M. javanica* populations according to their geographical region and host plants. The UPGMA trees generated using Dice coefficient for RAPD and ISSR are presented in Figures 4 and 5, respectively. We use bootstrap values upper 60 for separating main clades, and in other clades or subclades, bootstrap is under 60. Bootstrap values written only for main clades.

In analyses of molecular variance analysis, percentages of molecular variance within and among populations were 11.36% and 2.62% for RAPD, and 8.26% and 1.33% for ISSR, respectively (Table 6). *F*_*ST*_ and Nm for RAPD are 0.188 (P = 0.010) 1.079 however for ISSR are 0.140 (P = 0.010) and 1.535.

**Table 6.** Analysis of molecular variance (AMOVA) of *Meloidogyne javanica* populations based on RAPD and ISSR data.

Marker	Source of variation	Degree of freedom	Sum of squares	Variance components	Percentage of variation	Fixation index (*F*_*ST*_)^†^	Number of migrants (Nm)
	Between populations	6	148.88	2.63*	19		
RAPD	Within populations	30	340.85	11.36*	81	0.188	1.079
	Total	36	489.73	13.99			
	Between populations	6	90.68	1.34*	14		
ISSR	Within populations	30	247.80	8.26*	86	0.140	1.535
	Total	36	338.49	9.60			

**Note:** ^*^p < 0.01, ^†^*p* < 0.001

## Discussion

This is the first comprehensive study of RKN species in southern regions of Iran which demonstrated that *M. javanica* was the most predominant species of RKN infesting vegetable fields and greenhouses in southern Iran. Although there was a slight genetic variation at the intraspecific level, populations of *M. javanica* generally showed a low level of genetic diversity. In the present study, surveyed *Meloidogyne* species were characterized using morphological and morphometric characters and confirmed with species-specific SCAR-PCR. The genetic diversity among populations of *M. javanica* was further explored by ISSR and RAPD markers.

Accurate distinction of *M. enterolobii*, *M. javanica*, and *M. incognita* was still challenging using various molecular techniques, except for SCAR-PCR (Zijlstra et al., 2000) and genotyping by sequencing method for identifying diagnostic single nucleotide polymorphisms (Rashidifard et al., 2018). In our study, SCAR markers were used successfully for distinguishing populations of *M. javanica* from *M. incognita*, so 37 populations produced specific band of *M. javanica* and four populations produced the band typical for *M. incognita*.

On the whole, P, H, and I revealed a low level of genetic diversity within populations by incorporation of RAPD and ISSR data (Table 4). To our knowledge, this is the first study for the genetic structure of *M. javanica* populations revealed by ISSR marker. The RAPD and ISSR markers could not reveal any population-specific band and they failed to discriminate different populations of *M. javanica* by their geographical origin or plant hosts; which in agreement with those obtained by Adam et al. (2005) and Mokaram Hesar et al. (2011).

The mean *F*_*ST*_ and Nm values for the studied populations were 0.188 and 1.079 by RAPD while 0.140 and 1.535 by ISSR, respectively (Table 6), indicating that most of the total genetic variances (81 or 86%) were at the intra-population level. The greater the *F*_*ST*_ values are, the greater the differences between populations. *F*_*ST*_ and Nm are inversely related and when values of *F*_*ST*_ are low, values of gene flow are high, it means that a lot of gene exchange has occurred between populations. In nematodes and especially RKNs, the most important method of their distribution is through agricultural inputs; thus, populations could be distributed by infected transplants and other plant material. In this study, low values of *F*_*ST*_ and average values of gene flow may indicate some integration of populations with together. So higher values of genotypic variations within regions, rather than among regions, confirms this fact and that is the amount of genetic variation related to this species. The low genetic diversity observed for *M. javanica* isolates during the present study was similar to results reported for other populations of *M. javanica* from different crops (Carneiro et al., 1998; Randig et al., 2002; Cofcewicz et al., 2004; Medina et al., 2017). This finding might be attributed to the parthenogenetic mode of reproduction in *M. javanica*, which results in clonal progenies (Triantaphyllou, 1985). Measured individuals of the selected five populations of *M. javanica* in the present study could not be sorted in regard of none of the diagnostic characters (Table 3).

The amount of genetic diversity of RAPD marker in this study is higher than that of the northern regions of Iran, which has previously reported by Mokaram hesar et al. (2011), although the host plants are almost identical. Moreover, it seems that genetic diversity revealed by ISSR marker, is slightly higher; however, there is not any other reference that can be compared with the present results. One possible source might be related to the presence of males in the studied populations. Genetic changes in nematode population are partially driven by the environment including the use of resistant varieties and pesticides. Exploring such genetic changes at the population level is crucial for understanding and predicting the behavior of nematodes in the field (Golden and Birchfield, 1978). When we compare warmer conditions in southern regions with those in northern regions of Iran, it may be concluded that the crop cultivation is carried out consecutively throughout the year, and it seems that activity of RKNs continuing during the year, which may be a cause of increased genetic diversity among the present populations.

**Figure 6:**
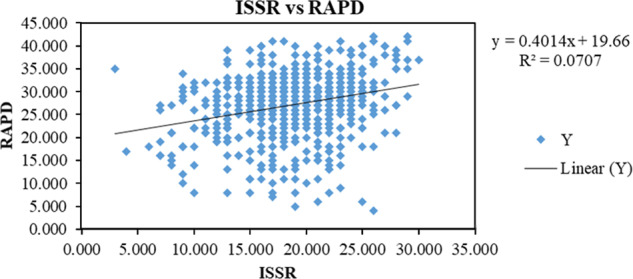
A Mantel test showing correlation between RAPD and ISSR datasets of the populations of *Meloidogyne javanica* recovered from southern Iran.

It should also be pointed that the results of Mantel test revealed that there is a low correlation between the two genetic diversity sets of RAPD and ISSR (*r* = 0.266, *P* ≤ 0.01), and it seems that these two markers may work independently (Fig. 6).

In this paper, the species-specific SCAR primer technique proved to be a powerful tool to distinguish the populations of the two widely distributed RKNs (*M. javanica* and *M. incognita*) from each other. The PCR-based techniques using RAPD and ISSR markers were shown to be helpful for obtaining detailed information about the diversity that exists among *M. javanica* populations. Perhaps including more populations from distant geographic regions, and even different countries, provide better understanding of the genetic structure and gene flow of these nematodes. Further intra- and inter-specific genetic diversity of *M. javanica* may be explored by implementing next-generation sequencing (NGS) techniques.
